# Making leisure time meaningful for adolescents: an interview study from Sweden

**DOI:** 10.1080/17482631.2023.2286664

**Published:** 2023-11-27

**Authors:** Katarina Bälter, Julia Johansson, Sara Karvonen Sheikh, Camilla Eriksson

**Affiliations:** a Division of Public Health, Mälardalen University, Västerås, Sweden; b Department of Medical Epidemiology and Biostatistics, Karolinska Institute, Stockholm, Sweden

**Keywords:** Adolescents, community actors, meaningful leisure time, qualitative, upper secondary school, health

## Abstract

Adolescents’ school performance is influenced by several factors and meaningful leisure time, especially organized activities, has great potential to impact academic results. Therefore, this study aimed to gain a greater understanding of how community actors perceive meaningful leisure time and how they work to create meaningful leisure time with the intention of increasing the chances for more adolescents completing upper secondary school. Semi-structured interviews with 14 informants, representing nine different community actors in a middle-sized city in Sweden, were conducted and analysed using content analysis. Results suggest that meaningful leisure time positively impacts adolescents’ mental health through social relations, support, and guidance. Leisure is believed to have spillover effects on reducing stress, manage school demands and performance. Nevertheless, leisure time activities and school performance must be balanced with time and effort. Community actors work proactively with availability, individual approaches, and offering activities to create meaning. From a societal perspective, places to hang out with supportive adults, in particular structured activities, should be regarded as a social investment in adolescents’ health and prospects, especially in deprived areas where fewer activities are available. Finally, ensuring meaningful leisure time is in line with the Convention on the Rights of the Child.

## 1. Introduction

Adolescents’ engagement and performance in school are influenced by factors both in school and outside of school. High academic performance has for years been positively associated with health determinants such as physical activity, good food and sleeping habits, parental support, and lack of mental illness (Brännlund et al., 2017; Ericsson & Karlsson, 2014; Gustafsson et al., 2010; Käll et al., 2014; Kark & Rasmussen, 2011; Lennernäs, 2011; Rothon et al., 2012; Russo et al., 2017; Taras, 2005; Tonetti et al., 2015). Moreover, creative and non-academic activities may also influence school performance positively by offering support from adults outside the family setting (Badura et al., 2016; Eccles et al., 2003). Organized group activities offer opportunities for improving self-confidence, developing leadership skills and social relationships, and preventing poor mental health. These positive effects may also impact school performance (Badura et al., 2021; Bean et al., 2019; Eime et al., 2013; Murphy et al., 2022). In addition, organized leisure activities are associated with better executive functions, compared with unstructured leisure time and extensive screen time (Ibabe et al., 2023). Yet, adolescents’ participation in organized leisure activities decreased in favour of unstructured activities during the last three decades. This event coincides with new legislation in Sweden in which grades were introduced into grade six in 2009 instead of grade eight, which has the potential to increase academic pressure at an early age (Lundvall & Thedin Jakobsson, 2021). More specifically, Behtoui (2017) shows that organized activities are positively associated with school performance, while unstructured leisure time has the opposite effect. Taken together, meaningful leisure time outside of school and overall good health have great potential to impact academic results positively.

Moreover, adolescents engaged in organized leisure time activities experience positive health benefits, regardless of family structure or socioeconomic status. However, adolescents living in non-nuclear families or low socioeconomic conditions are less likely to partake in organized leisure activities (Badura et al., 2021), which is also supported by Sukys et al. (2021) showing that high socioeconomic status or having high levels of health literacy is associated with higher physical activity levels.

In the past 20 years, education has become increasingly important for young people’s opportunities to enter the job market in developed countries (Swedish Association of Local Authorities and Regions, 2015). Sweden is no exception. One-fourth of adolescents in Sweden who enter secondary high school do not graduate (Swedish National Agency of Education, 2018), even though school is free of charge, free school lunch is offered every day, and, in most cases, a free computer is provided for schoolwork. Adolescents typically start secondary high school at age 16 and may choose among 18 national three-year programmes, both theoretical preparatory programmes for future university studies and practical programmes for skilled or certified trade jobs (Swedish National Agency for Education, 2019). National data show that completion of upper secondary school, even with low grades, increases the chances of employment by more than 50%, compared with not completing secondary high school for young adults at the age of 20 (Swedish Association of Local Authorities and Regions, 2015).

Adolescents with parents and caregivers who have low education, foreign backgrounds, or disabilities are less likely to graduate from secondary high school (Blomfield & Barber, 2011; Public Health Agency of Sweden, 2019b). In addition, the most common school-related reasons for not completing upper secondary education are insufficient pedagogical support in school; poor treatment in school; inadequate pedagogical support in school after a long-term absence; need for more practical and less theoretical education; disorganized school environment; incorrect programme selection, undetected neuropsychiatric disabilities; bullying and social exclusion; lack of adult support; and poor home conditions (Swedish Agency for Youth and Civil Society, 2013). Previous research by Hopper and Iwasaki (2017) suggests that adolescents with fewer resources and opportunities can benefit from meaningful leisure time, presenting opportunities to connect with friends on a positive note and promoting *“constructive meaning-making in their lives”.*

In the present study, leisure time includes everything except time spent at school, at work or sleeping. Hence, leisure time is synonymous with free time (Olson, 1993) and includes both structured and unstructured activities. Leisure time can invoke a subjective sense of freedom as the individual is free to choose how to spend time. Iwasaki (Iwasaki, 2017) has further tried to conceptualize leisure time as meaning-making, which he describes as created by strengthening life elements such as identity, creativity, connection, harmony, stress management, growth and experience, and existential aspects. In this sense, leisure time goes from an activity-based perspective to a more holistic perspective. Meaningful leisure time is anything that promotes a life that is joyful, connected, discovered, composed, and empowered (Iwasaki et al., 2018). Furthermore, the World Health Organization states that leisure time should be seen as a source of health improvement and important for adolescents’ ability to cope with the demands of everyday life and strive for a meaningful and balanced life (World Health Organization, 1986). Increased well-being through leisure time activities is therefore important for adolescents (Cosma et al., 2021; Murphy et al., 2022) and community actors who offer a range of activities and opportunities are key players in this context (Lindström, 2010). Moreover, Sweden has accepted the Convention on the Rights of the Child as law, which states that all children have the legal right to suitable and equal opportunities to partake in leisure and recreation, which shall be promoted by the member states (UNICEF, 1990).

Despite the positive effects of participating in organized leisure time activities (Bethoui, 2017), the number of adolescents who are members of or engaged in various community groups or organizations, such as sports clubs, music, and cultural schools, decreases from the age of 16 (Swedish Agency for Youth and Civil Society, 2014; The Youth Barometer, 2019). One explanation for this situation can be increased individual development and valuing their time, compared to earlier times when adolescents had a greater focus on the collective team success (Lundvall & Thedin Jakobsson, 2021). National data also show that adolescents with disabilities, living in neighbourhoods with low socioeconomic status, or with foreign backgrounds are active in or belong to community groups or organizations to a lesser extent than others and are more likely to visit local youth centres or libraries (Swedish Agency for Youth and Civil Society, 2014), compared to others. As adolescents from neighbourhoods with low socioeconomic status experience fewer opportunities to engage in local leisure time activities, and therefore miss out on opportunities for development, it is particularly advantageous for them to engage in leisure time activities (Blomfield & Barber, 2011).

The level of education has a major impact on future opportunities for becoming established in the job market, which in turn influences future socioeconomic status and health for the individual. An increasing number of young adults without upper secondary high school degrees leads to health inequalities on a social level (Diderichsen et al., 2012; Pini et al., 2018; Public Health Agency of Sweden, 2019b). In Sweden, leisure time activities take place outside of school and are often organized by different community actors, competing for the same resources (Lindström., 2011). In this study, “community actors” refer to those organizations, sectors, and actors who organize activities or support leisure activities in different ways: the library, religious centres, youth centres, and physical activities, among others. Therefore, the aim of this study was to gain a greater understanding of how community actors perceive meaningful leisure time and how they work to create meaningful leisure time with the intention of increasing the likelihood of more adolescents completing upper secondary school.

## 2. Methods

The study followed a qualitative approach that aims to gain a deeper understanding of a research area by collecting data based on a specific topic or experience (Thomas & Magilvy, 2011). A qualitative approach in line with a social constructivist worldview emphasizes the subjective version of the social reality (Creswell, 2009) here on community actors’ perception of upper secondary school students’ leisure time.

### 2.1 Sample and participants

The study was conducted in a medium-sized city in Sweden with a population of approximately 150,000 people, of which about 7,000 are adolescents 16–19 years old. The present study is part of a collaboration among partners in the Social Contract, *i.e*. a collaboration arena between local municipalities, regional health care organizations and the University, with the purpose of facilitating collaboration among researcher, local policy makers and practitioners. The informants were identified in collaboration with municipal officials from the city; hence it was a purposive sampling process to find informants who had employment or position in the County council, municipality, or NGO who were actively working with adolescents’ leisure time or school in a managerial, strategic, or operational way. Nine community actors were identified and all agreed to participate. These actors interacted with adolescents through sports, culture, youth centres, student-based initiatives in schools, religious centres, libraries, health care, and public health, upper secondary high school and students not qualified to start upper secondary schools, and students with incomplete grades. More information about the community actors can be found in Table I.

**Table I.** Overview of community actors.

Community actors	Type of organization	Number of Interviewees
Library	Municipal	2
Community actor supporting students with incomplete grades	Municipal	1
Youth centers (two different)	Municipal	3
Community actor promoting cultural activities for youths	Municipal	1
Community actor working with upper secondary high school and adult education	Municipal	1
Community actor who works with increasing safety in school and society	NGO partly run by municipal funds	2
Religious centre	NGO	1
Community actor promoting physical activity	NGO	1
Community actor responsible for health care and public health in the county	Politically governed	2

Informants represent the nine community actors presented above. Fourteen interviews were conducted in which nine informants worked at the operational level and five worked at the managerial level.

### 2.2 Data collection

Community actors received emails with information about the study and those who expressed an interest in participating received detailed information. The goal was to interview two informants by the same community actor to get a wider perspective and to increase credibility (Graneheim et al., 2017). However, this criterion was only met by four community actors. Interviews were conducted until we reached a point of saturation, in which no new information or categories emerged.

Individual interviews took place between June and October 2019. All interviews except one took place in private rooms at the informant’s workplaces and were not interrupted by any external circumstances. However, one interview took place in an open area at the informant’s workplace. During this interview, a colleague of the informant engaged in the conversation twice. This conversation may have influenced what the informant said. A semi-structured interview guide was used (Kvale & Brinkman, 2009). The questions referred to meaningful leisure time, health, and school performance and how the community actors work to create meaningful leisure time for adolescents. The interviews lasted between 40 minutes and 105 minutes; the median time period was 72 minutes. Interviews were audio-recorded. Some informants received the interview guide prior to the interview to prepare for the questions.

### 2.3 Data analysis

The interviews were transcribed verbatim and analysed by manifest content analysis (Graneheim et al., 2017). All interviews were conducted and transcribed in Swedish. Therefore, the analysis process continued in Swedish, and quotes were translated into English first before reading the manuscript.

The manifest content analysis distinguishes and highlights differences and similarities in a text and focuses on the visible and obvious elements that emerged. Meaning units, a constellation of words or sentences that can be related to the aim of the study, were identified and highlighted. Thereafter, each unit was marked with a code or label that described its content. Codes that shared a common meaning were sorted into subcategories and those subcategories were then divided into categories (Graneheim & Lundman, 2004). Naming and categorization were discussed and redefined until consensus was reached among J.J, C.E and S.K, who were mainly responsible for the analysis process. However, categories and subcategories were discussed and rearranged in the whole research group. The process is illustrated in Table II.

**Table II.** Illustration of the analysis process.

Meaning units	Codes	Sub-category	Category
… A leisure time that’s fun. And it can be a sport or something else like music, theater, or dance or whatever. That it makes you relax. You have fun, relax. Although it is tough for a while in school, you have a leisure time that is fun and that works for you. Or you have good and fun friends. […] But then also, I think leisure time should be pretty undemanding too. (IP4)	Fun and undemanding	The importance of balance and meaningfulness	Leisure time as a health-promoting resource
I can see that those who are going through tough times, fourteen, fifteen and are a bit lost and don’t have anyone. I mean, it’s not getting easier for them when they turn eighteen. It becomes like the youth center has been their lifeline. Here is where they hang out. The only place they spend more time at is their home. And even though we absolutely try to have it as a goal to help them go back to school … And we even changed our opening hours for that reason. (IP10)	Adapting activities	Identifying needs	Efforts to activate adolescents

### 2.4 Ethics

The study followed the ethical principles of research (Swedish Research Council, 1990) and is in line with the Declaration of Helsinki of ethical principles for medical research involving human subjects by the World Medical Association (2008). Informants consented to participate in the study after receiving written and verbal information about the study. The study reported on in this paper was approved by the Ethical Committee (dnr 2020–02133).

## 3. Results

The results are presented as two categories and seven sub-categories as presented in Table III below.

**Table III.** Results presented as categories and sub-categories.

Categories	Subcategories
Leisure time as a health-promoting resource	The importance of balance and meaningfulness
	Inequities in prerequisites for adolescents
Efforts to activate adolescents	Offering various accessible activities
	Identifying needs
	Individual-focused approach

### 3.1 Leisure time as a health-promoting resource

The first category describes how informants perceive meaningful leisure time. The informants described the importance of leisure time for adolescents’ health and school performance. Moreover, this section presents informants’ perceptions of how adolescents’ prerequisite may affect their chances of achieving meaningful leisure time.

#### 3.1.1 The importance of balance and meaningfulness

Leisure time was primarily perceived as a health-promoting resource that has a positive impact on both physical and mental health as well as on school attendance and education. Informants believed leisure time should be undemanding but fun and meaningful as described in the quote below. This time can include various activities such as physical recreation or culture.

… A leisure time that’s fun. And it can be a sport or something else like music, theater, or dance or whatever. That it makes you relax. You have fun, relax. Although it is tough for a while in school, you have a leisure time that is fun and that works for you. Or you have good and fun friends. […] But then also, I think leisure time should be undemanding too. (IP4)

Moreover, informants also stated that lack of meaningful leisure time can have negative consequences for adolescents. For example, adolescents’ negative social networks could lead adolescents to engage in criminal activities instead of participating in health-promotive activities. Therefore, community actors have a special focus on these adolescents to avoid destructive actions. One community actor who is often present in school environments describes:

… to monitor some adolescents that are at risk for criminality and so on […] and then you always try to steer them in another direction somehow, without putting others at risk. It is always necessary to make an assessment … (IP4)

Moreover, social pressure from peers may also result in poor school attendance as some prefer to go to the same school as their friends although the education does not suit them. This scenario is considered disadvantageous for adolescents’ school results.

The feeling of being good at something and being part of a group and physical activity was considered to promote qualities that may have an impact on learning ability, self-confidence, manageability, and the ability to structure schoolwork. The informants further believed that meaningfulness during leisure can benefit adolescents in their school performance.

When we do something, we enjoy, our brain gets a rest. The body can relax. If I am well rested, I go to school or work with joy. You have energy, you can manage. You get this driving force within you (IP9)

Say that you are doing something in your leisure time where you can be yourself a hundred percent. That can give you the strength to be yourself in an environment where you are not accepted as you are. Not like in the school, which is often so harsh and judgmental. (IP7)

Strengthening adolescents through leisure time could have a spillover effect on school and also lead to increased trust in society and in school staff, which was believed to increase school attendance.

#### 3.1.2 Inequalities in prerequisites for adolescents

The informants perceived differences in gender, age, and socio-economy in who participates in activities. For example, boys and adolescents in primary high school participate in the activities more than girls and adolescents in upper secondary school. This fact is explicitly mentioned regarding attendance in unstructured leisure centre:

With children, we have equal number of girls and boys attending here. But adolescents … The girls disappear … (IP8)

Girls were thought to be more negatively influenced by social media than boys. Older adolescents were perceived to be restricted in their leisure because most activities are organized for younger adolescents. Therefore, adolescents in upper secondary school may find it challenging to find meaningful leisure activities. Additionally, a decrease in participation in sports activities and an increase in competitiveness were perceived to make adolescents drop out of sports activities. Inequalities are further increased as some sports associations were considered to target adolescents with higher socioeconomic status and to exclude adolescents with low socioeconomic status, foreign origin, and disability. Therefore, there is an expressed need to reach those who become excluded from certain activities:

How do we get parents to see the importance of, or learn, how organized leisure works. How do we reach parents with information about what exists and what is done? […] How do we get to know what parents need? I think our dilemma is that we don’t reach the right target group … We have incredible competence inhouse, but we still don’t reach those who need it. (IP10)

The informants stated that school can affect adolescents’ leisure time. High demands and striving for high grades can result in less time for meaningful leisure time activities. It could also lead to school stress and mental illness. Adolescents were described as a time in life when they are finding their identity and becoming adults and simultaneously receptive to influences of peer pressure, macho culture, social media, and ideals.

To help achieve meaningful leisure time for adolescents, the informants stressed that activities and initiatives must be available. To promote accessibility and facilitate families with lower socioeconomic status, these activities should be in their neighbourhoods. The informants believed that a lack of associations and places where leisure time activities can be carried out may lead to alienation.

think that kids nationally but also locally, it’s possible to see kids that don’t, which live in more vulnerable areas have, they don’t experience their leisure time as meaningful as many others do. It can be that there aren’t many possibilities. If you live in a neighborhood with many apartments building and less green areas, they don’t have the same possibilities or don’t want to go out. Because they don’t feel safe, no cohesion. (IP5)

The informants further mentioned that parents and caregivers were important for adolescents to achieve meaningful leisure time. Caregivers with low socioeconomic status, those who suffer from illnesses, and caregivers with Swedish as a second language have limited opportunities to help their children achieve meaningful leisure time.

Not all parents have the competence or capacity to navigate through the activities that could actually be possible for their child. (IP9)

It’s important, what you get from home. And I think that’s more important than what you get, like, what you have during leisure. Although leisure is very important, and your context is very important. But your heritage. Culturally may be wrong … But education, what you inherit from your parents. (IP1)

In contrast, caregivers with a high standard of living could focus too much on performance. Informants believed that this could make adolescents develop mental illness and affect their leisure time despite socio-economic advantages. In addition, informants are starting to see another group of adolescents, from high socio-economic families who need support to achieve meaningful leisure time:

But what should we do with the group of adolescents who do not get the time […]? Because parents have yoga, running a marathon, making career. Have a summer house, boat, and new cars. What do we do with them? And these are the ones I think are starting to come. (IP2)

Insummary, informants from different community actors describe the importance of family support, economy, and engagement as well as differences in leisure activity due to gender and age. Hence, activities must be available and accessible for all adolescents, especially those who lack supportive structures in their families.

### 3.2 Efforts to activate adolescents

This category explains how community actors create leisure time for upper secondary school students with a focus on making it meaningful. This section also demonstrates what informants perceive to be important in their work to reach and activate adolescents, such as an individual-focused approach but also the importance of a physical place to be during leisure. Moreover, this category primarily represents perceived efforts towards adolescents who are not active in an organized activity and towards adolescents with different social challenges and living conditions.

#### 3.2.1 Offering various accessible activities

Community actors work with adolescents in different ways. Five actors are in direct contact with adolescents, aiming to increase security in school and society, and one organization supports students who have incomplete grades. The youth centres are often run by the municipality and are usually located in the vicinity of a school. Adolescents can meet at the youth centre after school and participate in both structured and unstructured activities such as cooking, playing ping-pong, or just hanging out.

I can see that those who are going through tough times, fourteen, or fifteen and are a bit lost and don’t have anyone. I mean, it’s not getting easier for them when they turn eighteen. It becomes like their, the youth center has been their lifeline. Here is where they hang out. The only place they spend more time at, is their home. (IP10)

Libraries usually offer activities for younger children and do not have targeted activities for upper secondary school students. However, some libraries have become meeting places for adolescents. The religious centre provides a meeting place for people of all ages, but they also have a section for adolescents where they decide what kind of activities they want. Four community actors work strategically and have an indirect effect on adolescents’ leisure time. One organization provides sports clubs with education and advises them on how to become more inclusive. They also encourage adolescents who otherwise are not engaged in sports or physical activity. They arrange events in which adolescents can try out different sports and have the member fee covered for a limited period. In addition, community actors make sure to provide places that adolescents can visit during their leisure in the evenings, on weekends, or on school holidays.

We focus on children and adolescents’ leisure, to increase the quality of leisure. And that means that there are different activities on weekdays and evenings. And a huge part of that is to coach adolescents. (IP 8)

We have projects in areas that are weak in sports where associations act during schooltime and initiate activities. We have tried to enable that, there is no threshold for children and adolescents to participate in these activities. (IP1)

Another organization responsible for public health issues conducts studies and writes health reports. These reports are forwarded to relevant community actors to improve and focus their activities, and to communicate research findings to policy makers so the priorities and financial support can target specific groups or issues. One informant elaborates:

How to create a meaningful leisure time? I think it’s about paying attention, that you can work to improve it. And how do you improve it and what does the research say and what does the adolescents themselves think is meaningful. I mean young people themselves know what is meaningful to them. And then we can use research to also learn more about the importance of a meaningful leisure time. If we know the significance of it (meaningful leisure time), then it is easier to argue that it should be prioritized, and resources can be deployed. That this is a factor that should be taken into account in various political or official decisions. And that’s probably how we can work with it, I think. To increase knowledge, awareness. Highlight young people’s perspectives through surveys and so on and to raise awareness. (IP6)

The community actor who promotes cultural activities for adolescents contributes to meaningful leisure time for adolescents by making it possible for them to arrange cultural events for other adolescents. Moreover, one school invites prospective students and their caregivers for a meeting where they talk about how caregivers can support their children during upper secondary school, for example by supporting them in their leisure time.

#### 3.2.2 Identifying needs

The informants stated that safe meeting places, such as youth centres, outside the home or school are important to achieve meaningful leisure time. Youth centres can have activities that provide meaningfulness to adolescents who for any reason are not enrolled in other activities. The community actors, therefore, believed youth centres should be given more resources.

Adjusting the opening hours of existing youth centres was suggested. This would mean being available for adolescents up to 18 and 20 years old by opening at the end of the school day. The importance of meeting places that are not related to a specific activity is further elaborated:

Some like to come here and some like to go to the youth center and some like to do sports. It serves a huge purpose for some. And some are better off in other places. So even though we do pretty much as the youth centers, I still think it feels like there is…it suits one target group. (IP14)

As stated in the quote, there must be different activities depending on the adolescents’ interests. Some adolescents create their meaningfulness in sports, others in a more relaxing and social activity. These meeting places are especially important and can have preventive measures in terms of health and education for those adolescents who experience challenges at home or in school.

Libraries receive many visits from upper secondary school students. These adolescents come there because they are too old to be at the youth centre or because they do not have anywhere else to be, not to read books and study. Informants pointed out that there are no meeting places available for older adolescents and young adults up to 25 years old.

There is no meeting place for adolescents after school. It’s a huge problem. Children who have attended the leisure center (for children) before comes back, but we can’t allow them to stay (when they get older) because we have children here during the days. […] It must be solved at another level […] we notice that there is a need for adolescents aged 14, 15 to have someplace to be, they don’t want to go home directly after school. But we have nothing to offer, only the streets of the city … (IP 8)

The need for unconditioned meeting places is to prevent adolescents from “hanging out in the city” and being more prone to engage in certain criminal activities. One informant spoke about what the premises for a meeting place for older adolescents could look like.

I think it would be cool if there was a house where you could lock the door and leave when you are done. […] Where you can decide. […] Because then there is a place that can be a physical place, that can also be the external communication in some way. (IP7)

In this meeting place for older adolescents, they should feel that they are responsible and own the premises, where they also can meet other young people with common interests. It is also suggested that they should be able to meet community actors who can provide information on topics such as financial maintenance, studies, or the labour market.

#### 3.2.3 Individual-focused approach

Informants expressed that meeting adolescents unconditionally, and genuinely, and paying attention to how adolescents are feeling made a big difference in building relationships with them during leisure activities. As the goal is to create good relationships with adolescents, it is perceived as important to be present, to really listen, to give positive feedback, and employ a coaching approach. Investing in the relationships, beyond the activity itself, is thought to avoid issues later in life for the adolescent, as described in the quote:

To support and guide is also needed. They don’t just need activities or to learn new things. There’s also a need for the preventative, promoting before it gets too serious. When life gets rough. (IP2)

Allowing adolescents to participate in the community actors’ work by giving them responsibility strengthens their development and inspires other adolescents to take initiative. However, informants mentioned that adolescents who are engaged also participate in several other leisure time activities. Consequently, some may not spend enough time on their schoolwork and might also stop coming to the activities. Adolescents who do not have a leisure time activity to go to were described to have restlessness and energy which they don’t know how to manage.

And I think there is, on the one hand, a need and frustration. But maybe neither the energy nor the ability to implement or they may not always know who to turn to or what to do. It is these young people who sit and hang around with us, these are the ones I am thinking of now. (IP12)

## 4. Discussion

This study highlights how community actors work to create meaningful leisure time and their perception of how meaningful leisure time might increase adolescents’ chances to complete upper secondary school. The findings show that informants perceived adolescents’ leisure time as a setting for health promotion (World Health Organization, 1986) especially through strengthening adolescents’ mental health in terms of happiness, positive relations, and growing as a person, *i.e.*, the hedonic well-being (Keyes et al., 2002). Informants also acknowledged adolescents’ leisure time as a place where adolescents may develop qualities that promote school performance. This is supported by research that shows the relationship between better school performance and health-promoting factors such as physical activity, eating habits, sleep habits, mental health, parental support, and participating in organized group activities (Badura et al., 2016; Bean et al., 2019; Brännlund et al., 2017; Gustafsson et al., 2010; Russo et al., 2017; Taras, 2005). Leisure time and leisure activities is a multidimensional concept, and the interaction between leisure and school performance is complex. One key finding in this study is that structured meaningful leisure time activities are perceived as an important context for strengthening adolescents’ mental health, which is assumed to affect school and school performance through its spillover effect.

Informants stressed that creating good relationships with adolescents by meeting them unconditionally and by showing genuine consideration as well as allowing adolescents to participate in the community actor’s work was considered important, as stated before (Lindström, 2010). This is in line with previous knowledge (Antonovsky, 1987); that opportunities to influence the own leisure time are regarded as valuable, as it led to increased manageability and meaningfulness. Youth leaders who provide social support enhance psychological well-being (Keyes et al., 2002) by comprehensibility, manageability, and meaningfulness for adolescents and helping them understand and overcome certain difficulties in life. The social support received in adolescents’ leisure time can help adolescents manage school stress and create an overall sense of coherence (Antonovsky, 1987). The informants believed that adults pay attention to adolescents’ psychological well-being during their leisure time and provide support for adolescents, especially those lacking support and guidance otherwise. This may further reduce the pressure on health care and may also provide economic benefits to the entire society.

Informants in this study perceived those adolescents from families with low socioeconomic status, with a foreign origin, and young people with disabilities, experience exclusion from associations and thus do not participate in associations to the same extent as other adolescents. Similar conclusions are found in the National reports (Public Health Agency of Sweden, 2017, 2019a; Swedish Agency for Youth and Civil Society, 2014; The Youth Barometer, 2019). Some parents are found to have limited abilities to help their children achieve meaningful leisure time, a situation that is also identified as a problem on a national level (Swedish Agency for Youth and Civil Society, 2014; The Youth Barometer, 2019). This is unfortunate, as leisure time activities contribute to adolescents’ development and are especially important as a health-protective factor for those with lower socioeconomic status (Baker et al., 2012; Blomfield & Barber, 2011). Strengthening the role of caregivers in creating meaningful leisure time for adolescents is an important practical implication and should be part of future efforts as strong family, parental support, and parental control help to improve children’s mental health and may increase the chances of better school results (Rothon et al., 2012). However, it would be valuable to investigate the impact of parental support, socioeconomic status, and participation in organized leisure activities respectively on school performance.

Informants perceived boys to be more involved in leisure times activities arranged by the community actors than girls. Prior research indicates that girls prefer more structured activities than just visiting youth centres (Geidne et al., 2016). This is reasonable in the present study as well, where most community actors offered unstructured activities where the majority of visitors were males. Moreover, available leisure time activities do not correspond to young people from low-status areas, young newly arrived immigrants, and young people with disabilities preferences (County council of Västmanland, 2017; Swedish Agency for Youth and Civil Society, 2014; The Youth Barometer, 2019). Prevention programmes and initiatives aimed at leisure time do not often involve adolescents’ perspectives on what makes leisure time meaningful (Hopper & Iwasaki, 2017). Given the sociodemographic development in Sweden, and other countries, this should be further investigated, to develop meaningful leisure activities that attract adolescents who are or have parents born abroad, including a wider intersectional perspective of ethnicity, disability, and gender.

Organized leisure time activities should be regarded as a social investment with the potential to break alienation (Nilsson, 2012) and to promote adolescents’ mental health. As described in this study, as well as others (Badura et al., 2016; Bean et al., 2019; Iwasaki et al., 2018; Murphy et al., 2022), leisure time should not be considered as “time away from studies”, but instead as recovery, time for building resilience, meaningful connection, and stress reduction. However, this positive effect seems to require some kind of structured activity, and not just passing time. For adolescents who are not engaged in sports activities or such, youth centres can be an organized health-promotive context (Geidne et al., 2016). Future research and policy-makers should focus on the adolescent perspective of meaningful leisure time, collaboration between community actors to achieve activities accessible for all adolescents, and the direct correlation between meaningful leisure and school performance. At last, both researchers and practitioners need to consider and strengthen the legal perspective on meaningful leisure in accordance with the Convention on the Rights of the Child.

### 4.1 Strengths and limitations

The study has some limitations. There are several other community actors working with adolescents outside the school in a similar field of work, as well as other professions at different levels who could have been included in the study. This may have produced a different result. On the other hand, qualitative methods are about discerning specific observations, and the sample was also done together with officials who had good knowledge of local community actors.

Leisure time is a broad concept and can be interpreted in different ways. This may have led to the informants’ experiencing difficulties in explaining meaningful leisure time and how they strive to create meaningful leisure time for adolescents. Some informants requested the interview guide prior to the interview to prepare for the questions, which may have contributed to further descriptions.

In order to strengthen dependability (Graneheim & Lundman, 2004; Graneheim et al., 2017), a co-researcher in qualitative methods was included in the analysis phase.

## 5. Conclusions

The community actors work to create meaningful leisure time for upper secondary school students by supporting them, providing a safe place for them to use in their leisure time, and arranging activities. Community actors’ work might lead to increased comprehensibility, manageability, and meaningfulness. Informants perceived meaningful leisure time as important for adolescents’ school performance. Empowering adolescents through meaningful leisure time could have positive effects on their health and school results and hence, result in fewer health inequalities. Working to achieve meaningful leisure time for upper secondary high school students could be part of a strategy of early social investments that enable adolescents to complete upper secondary high school and to live healthy and fulfilling lives.

## Supplementary Material

4.docx
