# Supplementary material for: Making leisure time meaningful for adolescents: an interview study from Sweden
Source: Int J Qual Stud Health Well-being. 2023 Nov 27;18(1):2286664. doi: 10.1080/17482631.2023.2286664 (PMC11000679; doi:10.1080/17482631.2023.2286664)
Supplement: 4.docx [file ZQHW_A_2286664_SM6656.docx]

**Katarina Bälter** serves as a Professor of Public Health at Mälardalen University since 2016. Her research mainly focuses on sustainable lifestyle and health from a public health perspective. Her research mainly takes on two major challenges; to reduce the incidence of diet-related disease and to combat climate change, and the research is grounded in the global sustainability goals. She has also been postdoc at Harvard School of Public Health and Boston University, and has been a research fellow at Stanford University.

**Julia Johansson** holds a one-year master’s in public health and worked as a research assistant in this project. Her expertise is in parent support.

**Sara Karvonen Sheikh** was a research assistant in this project. She holds a master’s degree in Global Health. Her education and work-life focus mostly on research and children and youth lifestyle.

**Camilla Eriksson** is senior lecturer in Public Health and holds a PhD in Social Work. Her research focuses on living conditions and determinants of health. In specific, her research has focus on young people with intellectual disability, digitalization, organization of welfare institutions participation, and mental health. She has extensive experience in qualitative methods and analysis.
